# Evaluation of arsenic, cobalt, nickel, lead, mercury, and titanium in goat blood and hair reared in Sicily and insights into blood biomarkers

**DOI:** 10.3389/fvets.2026.1748021

**Published:** 2026-02-04

**Authors:** Francesco Fazio, Vincenzo Nava, Fabio Bruno, Patrizia Licata, Claudia Giannetto, Giuseppe Piccione, Antonio Cannuli, Salvatore De Caro, Francesca Aragona

**Affiliations:** 1 Department of Veterinary Science, University of Messina, Messina, Italy; 2 Department of Engineering, University of Messina, Messina, Italy

**Keywords:** blood biomarkers, goat, hair, one health, trace elements

## Abstract

**Introduction:**

Exposure to harmful trace elements can lead to their accumulation in various biological substrates, resulting in the development of acute and chronic diseases in humans and animals. The knowledge of bioaccumulation of trace elements in different biological substrates could provide important information on exposure to trace elements through the use of sentinel animals. The aim of this research was to study the potential bioaccumulation of various trace elements (Arsenic-As, Cobalt-Co, Nickel-Ni, Lead-Pb, Mercury-Hg and Titanium-Ti) in goat blood and hair and their relationship with haematological biomarkers.

**Methods:**

Hair was collected from 25 non-pregnant and non-lactating clinical healthy goats aged between 2 and 3 years reared in Sicily, to determine trace elements concentration by means of inductively coupled plasma mass spectrometer (ICP-MS). Blood in duplicate was collected at the same time to determine trace element concentration and haematological profile. A paired *t*-test and multiple regression analysis was performed to evaluate the relationship among As, Co, Ni, Pb, Hg and Ti and blood and hair, together with their relationship with haematological parameters, respectively.

**Results and discussion:**

Statistical analysis showed a significant higher concentration of As (*p* < 0.0001) and Pb (*p* < 0.0001) in blood compared to hair and higher concentration of Co (*p* < 0.0001), Ni (*p* < 0.0001), Hg (*p* < 0.0001), and Ti (*p* < 0.0001) in hair compared to blood. Blood As and Ti showed a positive correlation with HGB (*r* = 0.48; *p* < 0.01; *r* = 0.52; *p* < 0.01). Blood Pb and Ti showed a positive correlation with HCT (*r* = 0.52; *p* < 0.01; *r* = 0.52; *p* < 0.01) and a negative correlation was observed for blood Co and WBC (*r* = −0.45; *p* < 0.01). The analysis of the results clearly indicates a strong correlation between the bioaccumulation of certain trace elements and some haematological parameters. These results should be examined thoroughly in goats to understand the significance of haematological factors in productive and reproductive activities.

## 1 Introduction

The assessment of trace element concentrations in animal biological matrices is an increasingly important field of research at the interface of animal health, environmental monitoring and human health—the so-called “One Health” paradigm, which recognizes that animal, human and ecosystem health are interconnected (1). Trace and toxic elements such as arsenic (As), lead (Pb), mercury (Hg), nickel (Ni), cobalt (Co) and titanium (Ti) may accumulate in terrestrial ecosystems from both natural and anthropogenic sources (e.g., mining, industrial emissions, agricultural inputs, grazing in contaminated soils). They could subsequently enter fodder, water, soil and ultimately animals and humans. Their toxicity is linked to their chemical characteristics, which mean that they are not biodegradable and tend to accumulate in internal organs, causing biochemical and pathological alterations in the blood (2, 3). They are able to evade cellular regulatory mechanisms and interact with native proteins, DNA and nuclear proteins, thereby suppressing their biological functions (4). The monitoring of such elements in livestock is thus relevant not only for animal welfare, but also for food safety, environmental risk assessment and sentinel-animal biomonitoring (5–9). Whole blood offers information regarding short-term exposure to toxic compounds; however, major keratinized structures like as hair, hooves, horns, tails, and manes are more advantageous due to their accessibility and the non-invasive, stress-free sample process. These structures represent an inert and chemically uniform source of heavy-metal bioaccumulation, signifying prolonged exposure to contaminants (10). Due to their significant dependence on environmental alterations, haematological variables may serve as useful indicators of metal pollution (11). It is compelling to investigate how different pollutants’ bioaccumulation and particular trace elements influence this species’ haematological response to anthropogenic stressors (12). Haematological biomarkers, identifiable by blood tests associated with the bioaccumulation of heavy metals, serve as valuable biological indicators for detecting alterations in the body’s homeostasis due to exposure to these metals. The value of blood biomarkers lies in their potential to reflect internal dose, early physiological effects (e.g., haematological or biochemical-metabolic changes) and thereby serve as indicators of exposure and potential harm (3).

In Mediterranean areas, the threat of trace-element accumulation is notably heightened due to climatic and soil features that affect metal mobility and durability. Seasonal drought, elevated soil erosion, and the historical coexistence of agricultural and industrial land uses result in a unique pattern of element accumulation (13). Mediterranean soils frequently exhibit heterogeneous metal distributions associated with prolonged anthropogenic influences, such as the application of phosphate fertilizers, livestock manure, and irrigation with poor water (14). Livestock rearing constitutes a notable and supplementary channel for the introduction and recirculation of trace elements throughout ecosystems. Intensive and semi-intensive husbandry systems depend on mineral supplements, veterinary medications, and designed feeds that may contain high concentrations of Cu, Zn, and occasionally Pb or Cd as inadvertent pollutants (15). In Sicily, animal production holds significant economic and cultural value, and its interplay with local environmental conditions presents particular challenges with trace element cycle. The island’s volcanic geology, along with historical mining operations and specific industrial zones, has resulted in regions where natural and human-induced trace element contributions combine. Research conducted in Mediterranean livestock systems demonstrates that grazing animals can function as reliable bioindicators of environmental contamination, especially in areas with intricate geochemical contexts such as Sicily (13). Furthermore, the conventional dependence on pasture-based systems, along with contemporary inputs like feed concentrates and mineral licks, heightens the necessity to oversee environmental exposure and residue accumulation in animal products.

In ruminants such as goats (species *Capra hircus*), trace element uptake occurs via ingestion of soil and vegetation, inhalation of dust and water intake. Animals serve as effective indicators of environmental contamination by hazardous metals (16–18). Prolonged exposure to heavy metals results in their heightened accumulation in diverse tissues, including hair and blood. Whole blood, serum, hair, and nails have been utilized to assess the levels of environmental contaminants in animal biomonitoring investigations (19, 20).

Moreover, hair has been used as a bio-indicator of environmental contaminants in goats undergoing alpine transhumance: the study found increases in hair arsenic concomitant with pasture exposure (12, 21).

Few studies have systematically measured the specific elements of interest (As, Co, Ni, Pb, Hg, Ti) in both blood and hair (or keratinous tissues) of goats, and even fewer have sought to link these concentrations to haematological and biochemical biomarkers (22–24).

This study seeks to assess the concentrations of arsenic, cobalt, nickel, lead, mercury, and titanium in the blood and hair of goats, evaluate correlations between these matrices, and investigate associations with blood biomarkers (RBC, WBC, Hb, Hct, PLT), thereby enhancing the practical application of goats as sentinel species for environmental and public health monitoring. The combination of trace-element analysis in goat blood and hair, alongside concurrent blood indicators, offers a potentially robust method for monitoring animal and environmental health. It endorses the One Health approach by connecting environmental pollution, livestock exposure, animal health impacts, and potential consequences for human and food chains.

## 2 Materials and methods

### 2.1 Ethical statement

All housing and care conformed to the standards recommended in the Guide for the Care and Use of Laboratory Animals and in Directive 86/609 EEC. The animal studies were approved by Ethical commettee from University of Messina (06/2023). The studies were conducted in accordance with the local legislation and institutional requirements for the protection of animals used for scientific purposes, national decree-law 113/2013 (2010-63-EU directive). Written informed consent was obtained from the owners for the participation of their animals in this study.

### 2.2 Study period and location

**Figure 1:**
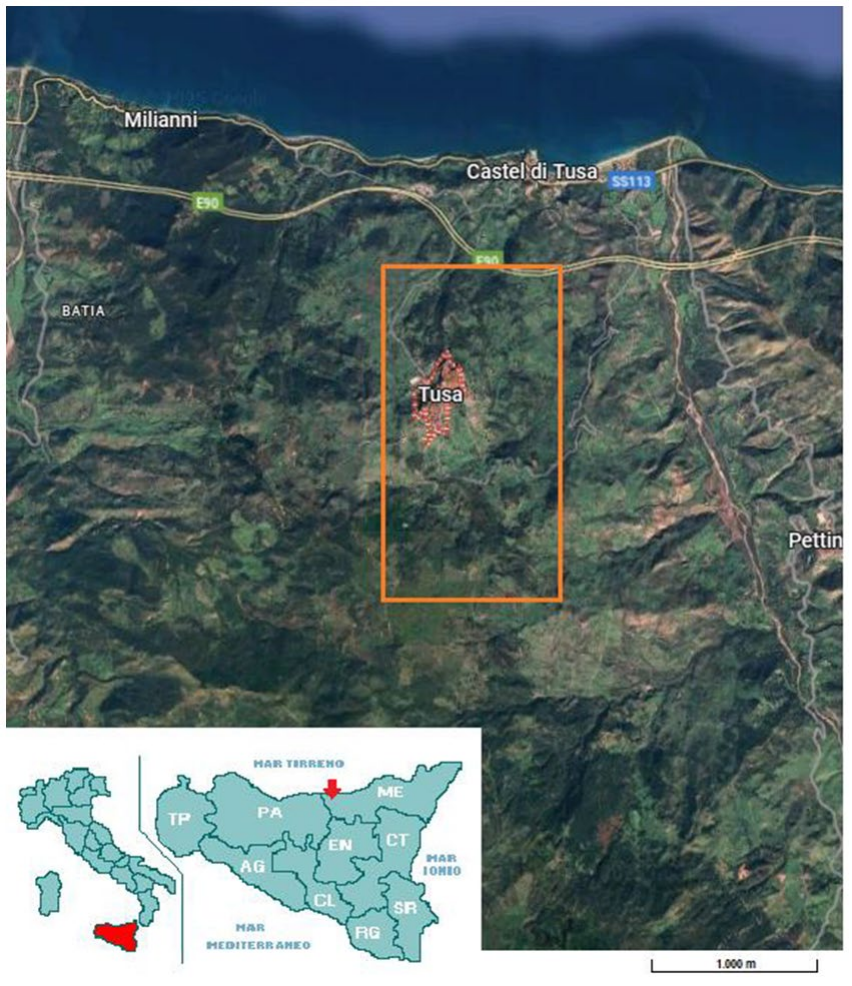
Map of Tusa, province of Messina, Sicily, Italy indicated with the red pointer. Orange square indicate the geolocation of farm belongs to the Nebrodi mountains as part of non-industrialised area of Sicily (Google Maps).

The present study was carried out in Tusa, province of Messina (Italy: 37°59′N 14°14′E), on a family-run farm from the Nebrodi mountains which is part of non-industrialised part of Sicily (Figure 1). Data collection took place in March 2025.

### 2.3 Animal selection criteria

Animals included in the study were previously selected based on a preliminary health status evaluation. Routine hemogram and physiological parameters such as rectal temperature (38–39.3 °C), respiratory rate (18–32 rpm), and heart rate (68–92 bpm) were monitored as inclusion criteria of the study. 25 female goat belonging to a Sicilian native breed, with a mean body weight of 30 ± 5 kg (2–3 years old) were enrolled in the present study. Not healthy subjects or subjects of different ages or who were pregnant or lactating were excluded, in order to obtain a homogeneous group in terms of age and physiological status among all subjects present on the farm. The animals were nourished on semi-extensive pastures comprising silt soil, hilly terrain, and a permanent polyphytic pasture consisting of meadow grasses and harvested grain crops, including grains and legumes on appropriate soils. The pastures consisted of perennial forage that self-seeded for a duration of 2 to 3 years. Preliminary monitoring of the animals’ physiological and haematological health, as well as their weight, indicated that no feed supplements were necessary, save during lactation in both species, so establishing that the grazing type was enough for the subjects assessed. The grazing area comprised native plant species and trees, including olive (*Olea europaea*) and chestnut (Castanea) groves. Animals were on free pasture with unrestricted access to feed and water.

### 2.4 Biological analysis

Biological samples were obtained under the oversight of the animal welfare manager and a veterinarian from the University of Messina. Each sample was performed in duplicate. Single hair sample (5 grams) were harvested from the rump region (30± mm) with plastic scissors to prevent contamination from extraneous minerals and subsequently preserved in plastic bags and stored until analysis at 4 °C. Blood samples were performed at 6 a.m. based on the circadian pattern of the haematological profile in mammals (25, 26). Blood samples were obtained in duplicate from jugular veins utilizing 3.5 mL sterile venoject tubes with ethylenediamine tetraacetic acid anticoagulant (K3EDTA, Terumo Corporation, Tokyo, Japan). One blood sample was used for haematological parameters determination (RBC, WBC, Hb, Hct, PLT) by means of an automated hematology analyzer (HeCo Vet C; SEAC, Florence, Italy) (2). The other blood sample was used for the determination of trace element concentration. For this purpose, a Thermo Scientific iCAP-Q inductively coupled plasma–mass spectrometry (ICP-MS) spectrometer ASX520 (Cetac Technologies Inc., Omaha, NE, USA) powered by a 27 MHz radiofrequency solid-state generator at 1,550 W was used. Linearity, accuracy (% recovery), and sensitivity in term of LOD (limit of detection) and LOQ (limit of quantification) were detected for each element using certified matrices (Whole Blood Metal Control Level 3, Milan, Italy, certified matrix) for blood and hair sample (ERM-DB001). For the determination of mineral element concentrations, a sample pre-treatment step was performed, consisting of a mineralization or acid digestion process. The sampling preparation including hair and blood followed specific procedure described in previous studies (1, 2, 27).

To ascertain the amounts of mineral elements, a sample pretreatment including mineralization or acid digestion was conducted utilizing a Milestone Ethos 1 microwave digestion system (Sorisole (BG)—Italy). Hair sample (0.5 g) was subjected to digestion using 7 mL of 65% nitric acid (HNO3) and 2 mL of 30% hydrogen peroxide (H2O2). The temperature was first elevated to 200 °C for 10 min at a microwave power of 1,200 W, thereafter maintained at 200 °C for an additional 12 min at the same power level. Blood sample (0.5 g) was utilized for mineralization. The reagent quantities consisted of 8 mL of 65% nitric acid (HNO3) and 2 mL of 30% hydrogen peroxide (H2O2). The operational parameters for this instance were as follows: (1) 2 min at 0–85 °C and 1,000 W; (2) 4 min at 85–135 °C and 1,000 W; (3) 5 min at 135–230 °C and 1,000 W; (4) 12 min at a constant 230 °C and 1,000 W. Subsequent to digestion, all samples were cooled for 20 min, diluted with ultrapure water to a final volume of 25 mL, filtered through 0.45 μm filters, and analyzed by ICP-MS. The analytical blanks and approved matrices were prepared identically to the respective samples.

During sampling, environmental parameters such as ambient temperature (21 °C), relative humidity (77%), and ventilation speed (3 km/h) were monitored using a multiparametric probe (Testo 400 SE & Co. KGaA, Titisee-Neustadt, Germany). Once the values had been read through the relevant measuring probes, the continuously recorded values were synchronized and analyzed through specific software (TESTO Data Control Vv 28.13.7) and displayed and reported as an average value. The monitoring of these parameters was crucial as environmental changes could directly impact health, metabolism, and survival.

### 2.5 Statistical analysis

All data were normally distributed (Kolmogo rov–Smirnov test *p* > 0.05). The statistical analysis was performed using Prism v. 9.00 (GraphPad Software, San Diego, CA, USA). All data were normally distributed (Kolmogorov–Smirnov test *p* > 0.05). A paired *t*-test was applied on As, Co, Ni, Pb, Hg and Ti results to describe possible differences between blood and hair elements concentration. Correlation analysis (Pearson) and the correlation coefficient (r) were investigated to observe the possible relationship among and substrates and the haematological parameters. *p* values < 0.05 were considered to be statistically significant.

## 3 Results

**Figure 2:**
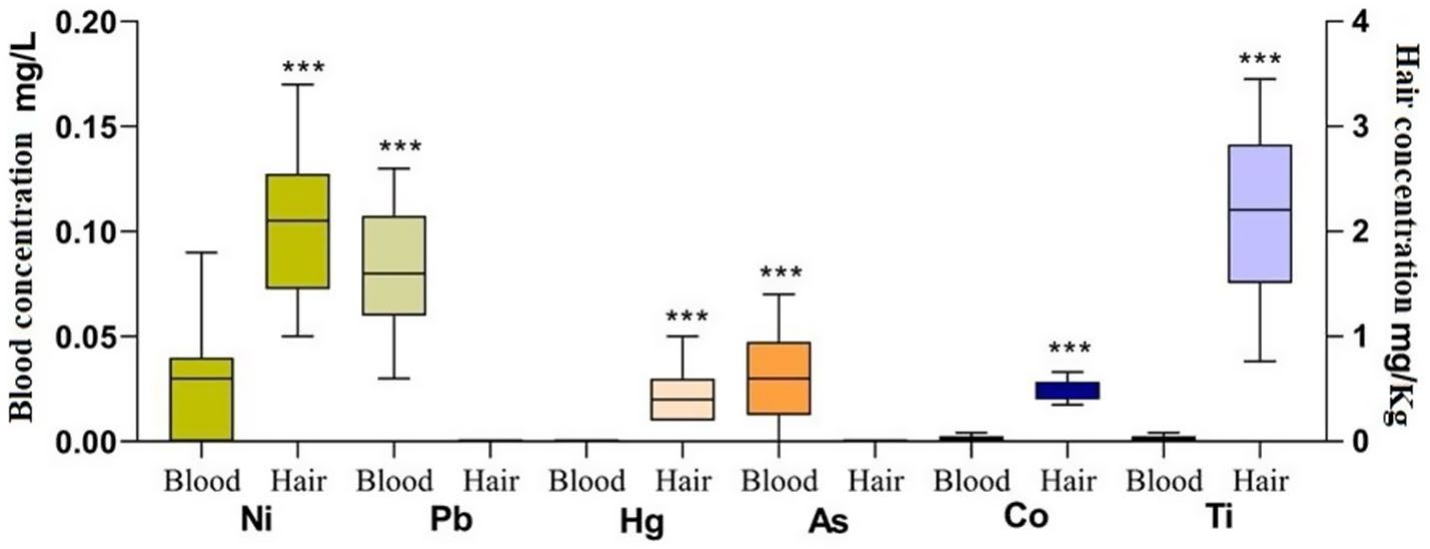
Mean ± standard deviation (SD) of Ni, Pb, Hg, As, Co, Ti concentration in blood and hair of goats (*n* = 25) expressed in their conventional unit (blood-left *y* axis and hair-right *y* axis). Different colors represent different mineral and for each mineral both substrates (blood and hair) were represented. ***Indicates the significances (blood vs. hair; *p* < 0.0001).

Different trace element concentration was reported as mean± standard deviation SD expressed in mg/L (blood samples) and mg/Kg for hair samples (dry weight) in Table 1. Paired *t*-test indicated a significant higher blood concentration of Pb (*p* < 0.0001) and As (*p* < 0.0001), compared to their hair level. A significant higher concentration of Ni (*p* < 0.0001), Hg (*p* < 0.0001), Co (*p* < 0.0001), and Ti (*p* < 0.0001) was observed in hair substrate compared to blood as shown in Figure 2. The blood concentration of As (*p* < 0.01) and Ti (*p* < 0.01) showed a positive correlation with HGB. The blood concentration of Ti (*p* < 0.01) and Pb (*p* < 0.01) showed a positive correlation with HCT. A negative correlation was observed for Co concentration in blood and WBC (*p* < 0.01) as shown in Table 2.

**Table 1:** Metal concentration ± standard deviation (SD) and min and max value minerals content in blood and hair substrate (mg/L−mg/Kg of dry matter).

Metals	Blood mg/L		Hair Mg/Kg	
	Mean ± SD	Min/max	Mean ± SD	Min/max
Ni	0.04 ± 0.02	0.02/0.09	0.10 ± 0.03	0.05/0.17
Pb	0.08 ± 0.03	0.03/0.13	*	*
Hg	*	*	0.02 ± 0.01	0.01/0.04
As	0.04 ± 0.02	0.01/0.07	*	*
Co	0.04 ± 0.02	0.02/0.08	0.49 ± 0.10	0.35/0.66
Ti	0.04 ± 0.02	0.02/0.08	2.16 ± 0.83	0.76/3.45

The symbol * indicates the concentration below the limit of quantification (LOQ).

**Table 2:** Correlation coefficients (*r*) of As, Ti, Pb, Co concentration observed among blood and haematological biomarkers (RBC, HCT, HGB, WBC and PLT) in goats (*n* = 25) including *p*-values and significances reported in bold.

Substrate	RBC		Hct		Hb		WBC		PLT	
	*r*	*p* value	*r*	*p* value	*r*	*p* value	*r*	*p* value	*r*	*p* value
BLOOD	As									
	0.34	0.14	0.43	0.05	**0.48**	**0.03**	−0.16	0.47	0.11	0.62
	Ti									
	0.27	0.24	**0.57**	**0.01**	**0.52**	**0.01**	−0.14	0.53	−0.21	0.36
	Pb									
	0.36	0.11	**0.52**	**0.01**	0.37	0.11	−0.07	0.74	0.05	0.68
	Co									
	−0.37	0.09	−0.11	0.65	−0.11	0.65	**−0.45**	**0.04**	−0.05	0.81

## 4 Discussion

The present study investigated the concentrations of different trace elements in blood and hair biological substrate, exploring their associations with haematological biomarkers (RBC, WBC, Hb, Hct, PLT) in Sicilian goats. The statistical comparison revealed that blood levels of Pb and As were significantly higher than the hair levels, whereas hair levels of Ni, Hg, Co and Ti were significantly higher compared to their respective blood levels. These findings illustrate differences in accumulation patterns of trace elements in small ruminants, and point to important considerations when choosing the biological substrate for exposure assessment. The higher blood concentration of Pb and As suggests that these elements may be more available in the circulating compartment (or perhaps more rapidly exchanged) than deposited in hair under the experimental conditions. By contrast, the higher hair concentrations of Ni, Hg, Co and Ti likely reflect longer-term accumulation in keratinous tissues. This pattern is consistent with the understanding that hair, hoof or horn matrices integrate exposure over longer time-frames and thus can capture cumulative or chronic exposure, rather than strictly acute uptake, whereas blood more closely reflects recent internal exposure (28).

Similar results of Pb and As were observed in a previous study conducted close to a mine tailings (29). The detection of these elements in blood samples validates the exposure of goats to trace elements, indicating that these elements were present in the bloodstream, potentially causing some detrimental effects on organs and the nervous system (30).

The considered trace elements, have been released into the environment and have interacted with living beings such as plants, animals and humans through various pathways. The present study did not reveal particularly worrying levels of trace elements in the substrates considered, based on previous literature on goats (24, 28, 29). It can therefore be assumed that the environment in which the animals lived represented mild and prolonged exposure to trace elements (29).

The present study demonstrates significant correlation between blood concentrations of certain trace elements (As, Co, Pb, Ti) and haematological parameters (RBC, WBC, Hb, Hct, PLT) in goats. These findings are fundamental for animal health, environmental monitoring and the broader One Health framework. The positive associations between As and Ti with HGB, and Ti and Pb with HCT may be associated with a compensatory erythropoiesis. The exposure to Arsenic (As) and Titanium (Ti) may stimulate the organism to increase RBC production, potentially in response to oxygen deficiencies. Similarly, Ti and Pb may enhance RBC production (Hct), wherein the bone marrow escalates RBC production elevating HGB and HCT due to stress or diminished oxygen-carrying capacity, possibly resulting from As/Pb interference with RBC function or oxygen utilization (31).

Chronic or low-level exposure to specific metals may induce a compensatory rise in erythropoiesis or hemoconcentration, resulting in elevated hemoglobin and hematocrit levels (31). Environmental exposure to trace elements may induces dehydration, fluid shifts, or reduced plasma volume, resulting in HGB and HCT elevation even in the absence of higher red-cell mass (32, 33). The positive connection observed between As and Ti with HGB/HCT could be attributable to the fact that arsenic can induce erythrocyte damage through oxidative stress, membrane impairment, and enzyme inhibition (34). An adaptive increase in erythropoiesis may arise, particularly in cases of mild hemolysis or hypoxia. Arsenic disrupts haem synthesis, oxygen transport, and red blood cell turnover (4). Titanium’s physiological effects in ruminants are not well understood. Titanium has been observed to accumulate in tissues and may affect oxidative stress pathways. The positive connection between titanium and HGB and HCT may indicate either a direct erythropoietic stimulus possibly considering goats grazing in titanium-rich soil encounter additional stresses (dust, dehydration), resulting in increased hematocrit and hemoglobin due to diminished plasma volume (35). The correlation between Pb and HCT warrants further consideration. Lead exposure is traditionally linked to anemia in ruminants due to the inhibition of hemoglobin production, heightened red blood cell disintegration, and reduced red blood cell longevity. However, if the exposure is relatively low and chronic, lead may induce subtle marrow stimulation or increased RBC turnover, or again the haematological effect may be confounded by concurrent stressors (eg dehydration, nutrition, sub-clinical inflammation) which increase HCT/HGB (36). The positive correlations between As/Ti and HGB, and Ti/Pb and HCT were somewhat in line with the concept that some trace elements can influence red cell mass or hemoglobin concentration, though directions vary by species and exposure context as observed in other studies in sheep (37).

In contrast, a negative correlation was observed between Co and WBC, where higher Co levels were observed to be significantly associated with lower WBC (38). This is consistent with the fact that Co imbalance can impair haematopoiesis (via its role in vitamin B12 metabolism) and thereby affect blood cell counts despite no multiple comparisons control (e.g., Benjamini–Hochberg FDR) has been performed. Moreover, some limitation of the work includes the small sample, the single location examined, the lack of soil and water evaluation. The present findings were opposite to previous findings in ruminants where lower Co correlating with rise in WBC (39). Our results could be attributable to bone marrow toxicity or displacement of normal trace-metal homeostasis leading to leukocyte suppression.

## 5 Conclusion

In conclusion, this study provides novel evidence that certain trace element concentration such as Arsenic, Cobalt, Nickel, Lead, Mercury, and Titanium observed in goat blood significantly correlate with haematological biomarkers. These associations underscore the potential of using goats as sentinel species in a One Health framework, linking environmental contamination to livestock exposure and haematological health biomarkers. While causal mechanisms remain to be clarified, the findings support further research into trace element monitoring in livestock, incorporation of haematological biomarkers into environmental health surveillance and linkage to food-chain safety. Moreover, the importance to investigate different biological substrates gives the idea of exposure of trace elements in both humans and animals as it reflects long-term accumulation with hair rather than short-term fluctuations seen in blood. Regular monitoring of haematological biomarkers with trace element concentration in biological substrates, could allow early detection of environmental contamination and animal sub-clinical health impacts improving animal welfare, ecosystem integrity and human health by early detection of environmental contamination.

## Data Availability

The raw data supporting the conclusions of this article will be made available by the authors, without undue reservation.
